# The burden of respiratory infections among older adults in long-term care: a systematic review

**DOI:** 10.1186/s12877-019-1236-6

**Published:** 2019-08-05

**Authors:** Arielle Childs, Andrew R. Zullo, Nina R. Joyce, Kevin W. McConeghy, Robertus van Aalst, Patience Moyo, Elliott Bosco, Vincent Mor, Stefan Gravenstein

**Affiliations:** 1 Department of Epidemiology, Brown University School of Public Health, Providence, RI USA; 2 Department of Health Services, Policy, and Practice, Brown University School of Public Health, 121 South Main Street, Box G-S121-8, Providence, RI 02912 USA; 3 Center of Innovation in Long-Term Services and Supports, Providence Veterans Affairs Medical Center, Providence, RI USA; 4 Sanofi Pasteur, Swiftwater, PA USA; 5 Faculty of Medical Sciences, University of Groningen, Groningen, NL the Netherlands

**Keywords:** Respiratory tract infections, Respiratory syncytial virus infections, Systematic review, Nursing homes, Frail elderly

## Abstract

**Background:**

Respiratory infections among older adults in long-term care facilities (LTCFs) are a major global concern, yet a rigorous systematic synthesis of the literature on the burden of respiratory infections in the LTCF setting is lacking. To address the critical need for evidence regarding the global burden of respiratory infections in LTCFs, we assessed the burden of respiratory infections in LTCFs through a systematic review of the published literature.

**Methods:**

We identified articles published between April 1964 and March 2019 through searches of PubMed (MEDLINE), EMBASE, and the Cochrane Library. Experimental and observational studies published in English that included adults aged ≥60 residing in LTCFs who were unvaccinated (to identify the natural infection burden), and that reported measures of occurrence for influenza, respiratory syncytial virus (RSV), or pneumonia were included. Disagreements about article inclusion were discussed and articles were included based on consensus. Data on study design, population, and findings were extracted from each article. Findings were synthesized qualitatively.

**Results:**

A total of 1451 articles were screened for eligibility, 345 were selected for full-text review, and 26 were included. Study population mean ages ranged from 70.8 to 90.1 years. Three (12%) studies reported influenza estimates, 7 (27%) RSV, and 16 (62%) pneumonia. Eighteen (69%) studies reported incidence estimates, 7 (27%) prevalence estimates, and 1 (4%) both. Seven (27%) studies reported outbreaks. Respiratory infection incidence estimates ranged from 1.1 to 85.2% and prevalence estimates ranging from 1.4 to 55.8%. Influenza incidences ranged from 5.9 to 85.2%. RSV incidence proportions ranged from 1.1 to 13.5%. Pneumonia prevalence proportions ranged from 1.4 to 55.8% while incidence proportions ranged from 4.8 to 41.2%.

**Conclusions:**

The reported incidence and prevalence estimates of respiratory infections among older LTCF residents varied widely between published studies. The wide range of estimates offers little useful guidance for decision-making to decrease respiratory infection burden. Large, well-designed epidemiologic studies are therefore still necessary to credibly quantify the burden of respiratory infections among older adults in LTCFs, which will ultimately help inform future surveillance and intervention efforts.

**Electronic supplementary material:**

The online version of this article (10.1186/s12877-019-1236-6) contains supplementary material, which is available to authorized users.

## Background

Acute respiratory infections cause approximately 4 million deaths per year globally [1]. In the United States, influenza, pneumonia, and respiratory syncytial virus (RSV) are projected to infect more than 13 million people annually, with an associated mortality burden of 69,000 to nearly 100,000 deaths attributable to just influenza in the 2017–2018 season alone [2–4]. Due to age-related characteristics like frailty and immunosenescence, respiratory infections produce more severe illness, a larger number of hospitalizations, and greater mortality in older than in younger adults [5, 6]. From the years 2012 to 2050, the size of the population of individuals ≥65 years is expected to double [7]. This growth creates a critical need to better understand the burden of respiratory infections in older adults [7].

Older adults residing in long-term care facilities (LTCFs) are an important subset of the older adult population that is at high risk of respiratory infections. Around 5% or more of persons ≥65 years are in LTCFs in developed countries, and LTCF use is rapidly growing in developing countries [8, 9]. These LTCFs house individuals in close quarters, an important consideration for contagious respiratory infections [10, 11]. The close proximity of residents in combination with advanced age, multimorbidity and frailty in LTCFs likely predisposes older adults to an even greater susceptibility to infections and their complications [12]. Furthermore, the clustering of frail older LTCF residents in close living quarters allows infections to spread more quickly. For diseases with morbidity and mortality that can be averted through vaccines, like bacterial pneumonia and influenza, low vaccination rates can contribute to the development of outbreaks [13]. Consequently, the LTCF setting and its residents are important targets for research and interventions [11].

Despite the common belief and plausibility that older LTCF residents are at a high risk of respiratory infections, to our knowledge, there has not been a rigorous synthesis of the existing evidence to empirically identify the burden of respiratory infections and guide future research or interventions. Nearly all prior studies and reviews have emphasized community-dwelling older adults, who have a lower prevalence of frailty and notably different characteristics. Much of the prior literature and its synthesis does not directly apply to LTCF residents. Synthesizing the evidence on LTCF residents can better inform future research as well as clinical and policy decision-making for this important population.

To address the need for evidence regarding the global burden of respiratory infections in LTCFs, we conducted a systematic literature review to summarize and appraise the current published literature.

## Methods

### Scope of the review

This systematic review was designed to understand the natural burden of respiratory infections (in the absence of vaccination or other treatment) among older adults in the LTCF setting.

### Data sources and searches

We systematically searched three databases (PubMed, Embase, and Cochrane Database of Systematic Reviews) for published systematic reviews on the prevalence and incidence of influenza, pneumonia, and RSV among LTCF residents in any country. Systematic reviews published in English between April 1964 and March 15, 2019 were considered. We individually evaluated (i.e., “handsearched”) the reference lists of the reviews to identify relevant articles to supplement our search for individual articles. We then systematically searched PubMed and Embase for relevant individual observational and experimental studies published in English during the same period. The database search strategies included the combination of terms in Additional file 1: Tables S1 and S2. Conference abstracts or proceedings were not included.

### Study selection

For individual articles, semi-automated abstract screening was performed using a machine-learning algorithm, which was trained by four reviewers to prioritize abstracts for screening from highest to lowest relevance (http://abstrackr.cebm.brown.edu) [14]. Abstracts for individual articles were independently assessed for inclusion by at least two reviewers.

We only included studies that specified participants to be aged ≥60 years, or that included a study population with a mean age ≥ 75 years and a standard deviation (SD) ≤ 6 years. These criteria reasonably ensured the study sample would be representative of a population aged ≥60 since > 95% of individuals could be expected to have an age ≥ 60 based on a normal distribution [15]. We also required that the study be conducted in a population residing in a LTCF setting that was not hospital-based because such hospital-based facilities typically operate more like an acute-care setting. To isolate the natural burden of infections in the absence of interventions and better understand the maximum potential impact that interventions might have when implemented alone or in combination, studies were only included if they provided data on an unvaccinated population which had not received either influenza or pneumococcal vaccine. Non-use of both influenza and pneumococcal vaccine was required due to the complex relationships between influenza and pneumonia (e.g., bacterial pneumonias in LTCFs may often result from viral infection that damages the lung epithelium to make it a rich culture medium for various bacteria), and the possibility that influenza vaccination may reduce the incidence of pneumococcal pneumonia. Similarly, we only included studies with populations not taking prophylactic antiviral medications for influenza (i.e., oseltamivir, zanamivir, peramivir, amantadine, and rimantadine). Studies examining RSV had no such requirements because there was no licensed vaccine for the virus at the time of this review. If a study did not describe vaccination or antiviral medication use and met all other criteria, we assumed the study population was unvaccinated or unexposed to antiviral medications and included the article. We did not evaluate studies for inclusion based on the method by which a respiratory infection was characterized because the various methods were all considered valid. As an example, viral testing is uncommonly done to diagnose influenza as clinicians often make a clinical diagnosis based on symptoms, judgement, and local influenza activity [16]. Likewise, bacterial pneumonia is also typically a clinical diagnosis and, in the LTCF setting, empirically treated. It is noteworthy that the various causes of pneumonia are essentially clinically indistinguishable from one another and the likelihood of a correct diagnosis outside of systematic testing is increased when there is a laboratory-confirmed case in the context of a cluster of individuals becoming ill around the same time (i.e., an outbreak).

In addition to studies describing the prevalence and incidence of influenza, pneumonia, and RSV, we were also interested in including literature on the outcomes and costs of such infections in LTCFs. Studies were excluded if they were case reports, editorials, case series, or commentaries. We adjudicated disagreements about exclusions through discussion.

### Data extraction and quality assessment

Data were extracted into the Systematic Review Data Repository (https://srdr.ahrq.gov). For each included study, one reviewer (A.C.) extracted study characteristics and information on respiratory infection occurrence, including whichever measures of occurrence were available or calculable--incidence proportion, prevalence proportion, incidence rate, and prevalence rate. Study characteristics included study design, geographic location, study dates, inclusion and exclusion criteria specific to the study, and whether the study focused on an infection outbreak or not. Data about the study population were also extracted, such as risk factors for infection and mean age (and whenever possible, SD). In studies of an intervention that could affect the risk of infection, data was only extracted from the control group.

### Data synthesis and analysis

Study data were synthesized qualitatively. Considering the relatively small number of studies, inconsistencies between studies, differences in outcome measures, and heterogeneity of the LTCF populations between countries, we elected not to perform a quantitative synthesis. Due to the nature of the review questions, we also elected not to perform a strength of evidence assessment.

## Results

### Literature search

**Fig. 1:**
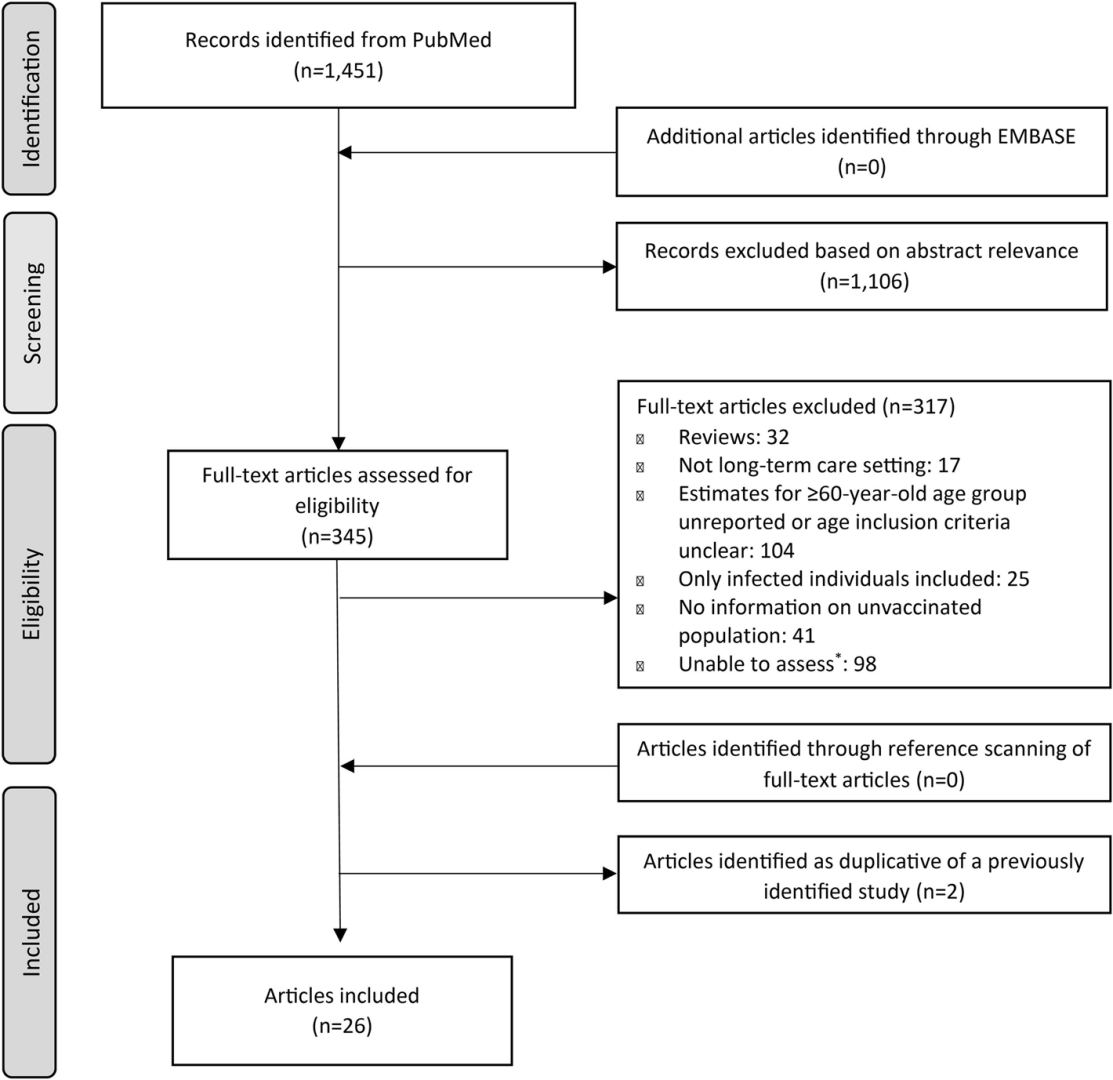
Flow of the selection process for literature included in the review. Asterisk symbol (*) in Figure indicates that articles could not be evaluated because they were in not written in English; the full text could not be accessed; or measures of occurrence were not presented and there were insufficient data to calculate measures of occurrence for infections of interest

Our search for existing systematic reviews yielded 46 results. For the search of individual articles, we screened 1451 citations (Fig. 1). Of those, 345 were selected for full-text review. No additional articles were identified for inclusion from Embase after searching PubMed.

### Overall study characteristics

We included 26 studies: 3 on influenza, 7 on RSV, and 16 on pneumonia. Fifteen studies were from North American countries, 6 from Asian countries, and 5 from European countries. There were 11 prospective cohort studies [17–27], 10 retrospective cohort studies [28–37], 3 randomized controlled trials (RCTs) [38–40], and 2 cross-sectional studies [41, 42]. The mean age of the study populations ranged from 70.8 to 90.1 years among all but 6 studies that reported a mean age. Male sex ranged from 16.5 to 54% of study participants, with no data for 8 studies. The time of data collection ranged from February 1979 to “winter 2014”, with no information on time of data collection for one study. The range of study sample sizes was 52 to 102,842 subjects and follow-up period lengths ranged from 30 days to 4 years. Two studies were industry-funded, 17 were not industry-funded, and the remaining 7 did not report the source of funding. Of the included studies, 18 reported an incidence estimate while only 7 reported a prevalence estimate; one study reported both. No literature was identified on the outcomes or costs of developing a respiratory infection in LTCFs.

### Influenza

Three studies provided data on the natural burden of influenza among LTCF residents (Table 1). Two were retrospective cohort studies and one was an RCT. All three studies were conducted before the year 2000. Two studies specifically reported on outbreaks.

**Table 1:** Published Literature on the Burden of Influenza in Long-term Care Facilities

Author, Publication Year	Study Design	Country	Study Dates	Type of study	Age, Mean (SD)^a^, in years	n Infected / N at Risk^b^	Incidence Estimates
Centers for Disease Control and Prevention, 1983 [28]	Retrospective Cohort	United States	December 1, 1982 - January 4, 1983	Outbreak	86.4	23 / 27	85.2%
Horman et al., 1986 [29]	Retrospective Cohort	United States	December 8, 1980 - January 13, 1981	Outbreak	83.2 (range 62–100)	*Influenza Definition 1—*Chest congestion/cough and temperature ≥ 37.8 °C: 12 / 59; *Influenza Definition 2—*Chest congestion or cough or temperature ≥ 37.8 °C: 28 / 59	*Definition 1—*20.3%; *Definition 2—*47.4%
Deguchi et al., 2000 [38]	Randomized Controlled Trial	Japan	November 1998–March 1999	Non-outbreak	81.4	694 / 11,723	5.9%

^a^If available and unless another measure is specified

^b^The estimates and study participants’ characteristics were calculated among the unvaccinated individuals

One of the retrospective studies focused on an outbreak of influenza A in a US LTCF with 170 residents, of which 59 were unvaccinated. Individuals were assessed and grouped according to two different definitions of infection. The first definition (definition 1) identified individuals with more severe symptoms (high fever *and* either chest congestion or cough). The second (definition 2) identified individuals with milder symptoms (high fever *or* chest congestion or cough). Definition 1 resulted in an incidence of 20.3% and definition 2 an incidence of 47.4% [28]. The other US retrospective cohort study also focused on an influenza outbreak and reported an incidence of 85.2% among 27 unvaccinated LTCF residents [29].

The third influenza study was an RCT that investigated the efficacy of the influenza vaccine among older adults in Japanese LTCFs [38]. Within the control group of 11,723 unvaccinated LTCF residents, the incidence of influenza was 5.9% [38].

No studies provided prevalence measures. The small number of influenza studies precluded examining patterns by geography, study design, or other study characteristics.

### Respiratory syncytial virus

The incidence of RSV among LTCF populations was evaluated in four prospective cohort studies, two retrospective cohort studies, and one RCT (Table 2). Among these seven studies, six reported incidence proportions and one reported an incidence rate.

**Table 2:** Published Literature on the Burden of Respiratory Syncytial Virus in Long-term Care Facilities

Author, Publication Year	Study Design	Country	Study Dates	Type of study	Age, Mean (SD)^a^, in years	n Infected / N at Risk^b^	Incidence Estimates
Sorvillo et al., 1984 [30]	Retrospective Cohort	United States	February–April 1979	Outbreak	79.2	13 / 101	12.9%
Ellis et al., 2003 [31]	Retrospective Cohort	United States	August 1, 1995 - July 31, 1999	Non-outbreak	Age ≥ 65, 100%	1105 infections / 88,851 person-years^c^	12.4 cases per 1000 person-years
McElhaney et al., 2004 [39]	Randomized Controlled Trial	United States	2000–2001^d^	Non-outbreak	82.2 (8.4)	3 / 198	1.5%
Caram et al., 2009 [18]	Prospective Cohort	United States	January 29, 2008 - February 26, 2008	Outbreak	70.8 (15.0)	7 / 52	13.5%
Johnstone et al., 2014 [17]	Prospective Cohort	Canada	2009, 2010, 2011^e^	Non-outbreak	Median 86 (IQR 80–90)	12 / 1072	1.1%
Uršič et al., 2016 [19]	Prospective Cohort	Slovenia	December 5, 2011 - May 31, 2012	Non-outbreak	Median 84 (IQR 79.8–88.8)	5 / 90	5.6%
Hui et al., 2008 [20]	Prospective Cohort	China	April 2006–March 2007	Non-outbreak	84.9 (8.9)	21 / 194	10.8%

^a^If available and unless another measure is specified

^b^The estimates and study participants’ characteristics were calculated among the unvaccinated individuals

^c^Only the number of person-years contributed by study sample was reported, not the number of individuals in the sample

^d^The published paper stated that study participants were enrolled in September or October and followed to the end of the respiratory viral season

^e^Participants were enrolled from mid-February through mid-March in 2000 for the first influenza season and trial. Then, a second trial was *initiated* in late December 2000 during a second influenza season, but the time period of enrollment was not reported

Five measures of occurrence were from non-outbreak studies, with incidence proportions ranging from 1.1 to 10.8%, and an incidence rate of 12.4 cases per 1000 person-years [17, 19, 20, 31, 39]. The two studies focused on outbreaks reported higher incidence proportions of 12.9 and 13.5% [18, 30].

No studies provided prevalence measures. Incidence did not appear to differ markedly by geography or study design.

### Pneumonia

Seventeen studies reported incidence and prevalence data for pneumonia in LTCF populations (Table 3). We identified seven prospective studies, six retrospective studies, two cross-sectional studies, and one RCT.

**Table 3:** Published Literature on the Burden of Pneumonia in Long-term Care Facilities

Author, Publication Year	Study Design	Country	Study Dates	Type of study	Age, Mean (SD)*, in years	n Infected / N at Risk†	Estimate of Occurrence
Cartter et al., 1990 [33]	Retrospective Cohort	United States	December 1, 1984 to April 10, 1985	Outbreak	Median: Nursing Home A: 84 Nursing Home C: 85	Nursing Home A: 3 / 16 Nursing Home C: 25 / 126	Nursing Home A Incidence Proportion: 18.8% Nursing Home C Incidence Proportion: 19.8%
Langmore et al., 2002 [41]	Cross-sectional	United States	1993–1994	Non-outbreak	Age ≥ 85, 49.4%; Age 65 to 84, 51.6%	3118 / 102,755	Prevalence: 3.0%
Konetzka et al., 2004 [34]	Retrospective Cohort	United States	1996	Non-outbreak	83.5 (7.4)	766 / 5899	Prevalence Proportion: 13.0%
Quagliarello et al., 2005 [21]	Prospective Cohort	United States	February 2001 to March 2003	Non-outbreak	84.7 (8)	112 / 613	Incidence Proportion: 18.3%
Won et al., 2006 [35]	Retrospective Cohort	United States	June 1998 to December 2000	Non-outbreak	83.4‡	75 / 3547	Prevalence Proportion: 2.1%
Givens et al., 2010 [22]	Prospective Cohort	United States	2003 to 2009	Non-outbreak	86 (7.0)	133 / 323	Incidence Proportion: 41.2%
Aparasu et al., 2013 [36]	Retrospective Cohort	United States	July 1, 2001 to December 31, 2003	Non-outbreak	83.5 (8.1)	Atypical Antipsychotic‡: 295 / 3609 Typical Antipsychotic‡: 188 / 3609	Atypical Antipsychotic Incidence Proportion: 8.17%; Atypical Antipsychotic Incidence Rate‡: 4.61 cases per person-year; Typical Antipsychotic Incidence Proportion: 5.21%; Typical Antipsychotic Incidence Rate‡: 5.21 cases per person-year
Huybrechts et al., 2011 [32]	Retrospective Cohort	Canada	January 1, 1996 to March 31, 2006	Non-outbreak	83.8 (6.9)§	Prevalence: 920 / 10,900; Incidence: 265 events / 2890 person-years	Prevalence Proportion: 8.4%; Incidence Rate: 9.17 per 100 person-years
Sund-Levander et al., 2007 [23]	Prospective Cohort	Sweden	2000–2003	Non-outbreak	84.6 (6.7)||	44 / 234	Incidence Proportion: 28.9%
te Wierik et al., 2012 [24]	Prospective Cohort	Netherlands	January to March 15, 2010	Outbreak	90.1 (1.1)	9 / 140	Incidence Proportion: 6.4%**
Rummukainen et al., 2013 [43]	Cross-sectional	Finland	2011	Non-outbreak	Age > 85, 49%**	75 / 5262	Prevalence Proportion: 1.4%
Sarabia-Cobo et al., 2016 [25]	Prospective Cohort	Spain	2011–2013	Non-outbreak	88.7 (6.8)	1330 / 2384	Prevalence Proportion: 55.8%
Fukushima et al., 2008 [26]	Prospective Cohort	Japan	December 1, 2003 to March 28, 2004	Non-outbreak	85	17 / 284	Incidence Proportion: 6.0%
Wu et al., 2010 [40]	Randomized Controlled Trial	Taiwan	2004	Non-outbreak	82.3 (8.3)	No data / 74	Traditional Model Incidence Rate: 0.17 cases per 1000 bed-days; Integrated Care Model Incidence Rate: 0.07 cases per 1000 bed-days††
Doi et al., 2014 [37]	Retrospective Cohort	Japan	Winter 2014	Outbreak	81.5 (8.5)	5 / 99	Prevalence Proportion: 5.1%
Kikutani et al., 2015 [27]	Prospective Cohort	Japan	No data	Non-outbreak	86.7 (7.8)	33 / 691	Incidence Proportion: 4.8%

*If available and unless another measure is specified

†The estimates and study participants’ characteristics were calculated among unvaccinated individuals whenever both vaccinated and unvaccinated individuals were included in the study samples

‡Rates were only provided by atypical versus typical antipsychotic exposure status

§Calculated by combining individual groups’ data using methods recommended by the Cochrane Collaborative

§Calculated from 1-year follow-up

||Measured during the initial 5 weeks of the study period

**Age derived only from the nursing home residents

††Rates were only provided for each arm of the trial

The majority of studies were conducted in non-outbreak settings with incidences ranging from 4.8 to 41.2%. Prevalence estimates ranged from 1.4 to 55.8% [21–23, 25–27, 32, 35, 41, 42]. Incidence rates were 0.07 and 0.17 cases per 1000 bed-days, as well as 9.17 cases per 100 person-years [32, 40]. Incidence rates of 4.61 and 5.21 cases per person-year were reported for atypical and typical antipsychotic users, respectively [36]. The incidence proportions were 8.17 and 5.21%, respectively [36]. Among the three outbreak studies, the two incidence estimates were 6.4 and 19.8%, and the prevalence estimate was 5.1% [24, 33, 37].

The burden of pneumonia did not appear to markedly differ by measure (incidence or prevalence), outbreak versus non-outbreak, or US versus non-US geography.

A single study reported risk factors for incident pneumonia in an unvaccinated study population [27]. These risk factors included activities of daily living status and the presence of swallowing disorders, ischemic heart disease, or dementia [27].

## Discussion

Our systematic review of published literature reporting on the natural burden of influenza, RSV, and pneumonia among older adults in LTCFs included 26 studies with highly variable estimates ranging from 1.21 to 85.2% for incidence and 1.4 to 55.8% for prevalence across all infections. Despite the variability in the estimates, these data suggest that the incidence and prevalence of respiratory infections are high among older LTCF residents. The available data underscore the lack of nationally representative, modern, and large studies of the burden of respiratory infections in this important population. While studies are necessary for all respiratory infection types, studies are particularly necessary to better understand the epidemiology of influenza among LTCF residents. Without such well-designed studies to inform interventions, clinical practice, and policy, an evidence-based approach to the reduction of the burden of respiratory infections in LTCFs will be unnecessarily challenging.

This systematic review uncovered several important themes of the underlying literature on the burden of respiratory infections in LTCFs.

Nearly all studies had small sample sizes of not more than a couple hundred LTCF residents, suggesting that estimates of the incidence and prevalence are unlikely to generalize broadly. Therefore, the selection of epidemiologic studies to inform clinical or policy decision-making, intervention development or implementation, and other initiatives to intervene on infection risk in LTCFs must consider sample sizes and study designs.

Studies of risk factors for incident respiratory infections in LTCFs were surprisingly scarce. Identifying predictors and developing tools to evaluate the respiratory infection risk of patients should be a focus of additional research. Existing datasets like linked Medicare claims and Minimum Data Set assessments could be used to identify predictors in routinely collected data. The increasing availability of electronic medical records in LTCFs could then support individual patient models for specific infections. For instance, once predictors have been empirically identified, electronic medical record data could be used to output the 6-month probability of developing pneumonia for each patient. Akin to the lack of studies examining predictors, no studies meeting our search criteria attempted to quantify the effects of incident respiratory infections on outcomes like healthcare costs, hospital readmissions, disability, or mortality. While respiratory infections certainly have a negative impact on all such outcomes, a better understanding of the magnitude of those effects and how they vary across subgroups of LTCF residents would be valuable for developing and implementing interventions to reduce the burden of respiratory infections.

The literature on respiratory infections presented several challenges to interpretation. Seven articles included in this review focused on outbreaks, which give rise to estimates that are likely higher than typically observable in LTCFs. The definition of an outbreak also varies markedly across studies by geography, type of respiratory infection, population involved, time, and more [43]. Many outbreaks are generally defined as an infection incidence exceeding an expected rate. Interestingly, few data are available to quantify or provide guidance about what an accurate and reliable estimate of an “expected rate” is. Studies reporting higher incidence rates (e.g., > 15%) may actually be outbreak studies even if they were not labeled as such. Converse to the potential overestimation of infection rates by outbreak data, data sources like administrative claims capture only some of the most severe infections and likely underestimate the burden of respiratory infections. Administrative data may identify even fewer respiratory infection events among LTCF residents because signs and symptoms of acute infection are often not proportional to the severity of illness [43]. Interpretation of the literature is further complicated by the atypical presentation of infections in frail older adults [44]. For example, LTCF residents may have pneumonia without cough, chest pain, or fever; tachypnea or confusion may be the only indicators [45, 46]. Atypical presentation is even more likely among those who are very old, cognitively impaired, multimorbid, or frail. The atypical presentation of respiratory infections in LTCFs can therefore lead to underdiagnosis and artificially low prevalence and incidence estimates.

The majority of the included studies reported respiratory infection incidence data and most were from North America. The focus on North America may have resulted, in part, from our focus on LTCFs unassociated with a hospital and the differences in LTCF structure or care systems between geographic regions or countries [47]. Places other than North America have many hospital-based LTCFs or have few LTCFs due to the use of alternative care settings. If LTCFs in North America have more resources than LTCFs in other geographic regions, the large number of North American studies could meaningfully impact estimates. LTCFs with more resources are more likely to have physician extenders (e.g., nurse practitioners, physician’s assistants) and larger amounts of staff to help implement interventions to reduce respiratory infections. LTCFs with more resources may also be more likely to have private rooms, which are better at limiting or containing the spread of infections. However, the incidence of RSV did not appear to differ between studies conducted in North America versus elsewhere. In aggregate, the burden of pneumonia also did not differ markedly between North America and other places, but when stratified on measure of occurrence, the incidence was higher in North America while the prevalence was higher in countries outside North America. Future studies should examine the influence of LTCF resources and characteristics on respiratory infection rates.

Our findings support the idea that healthcare providers should promote immunization against influenza and pneumonia, and rigorously implement prevention practices to avoid the spread of infections, particularly in unvaccinated populations [48]. Vaccination rates in LTCFs have been slowly increasing over time, but many residents remain unvaccinated and considerable improvement in vaccination rates is possible [49]. Of relevance, the small number of influenza studies precluded a meaningful analysis of time trends for influenza in our review, but no linear or other time trends for pneumonia or RSV were apparent, even after stratifying on location of study (North America versus not), type of study (outbreak or non-outbreak), or estimate of occurrence (incidence or prevalence). Our results suggest that a rigorous examination of how time trends in vaccination rates have impacted time trends in respiratory infections may be necessary to understand the absence of a relationship between the two across included studies. The results of our review also suggest that policy-makers should consider LTCF-specific policies to improve uptake of preventive interventions for reducing the burden of respiratory infections [48].

Our systematic review findings must be interpreted in light of several potential limitations and considerations. One potential limitation of the review is the search restrictions we introduced to focus the review. For example, we only included articles that were published in English. Although we included studies from several geographic locations, language barriers may have resulted in the exclusion of eligible studies of highly unvaccinated populations.

A second potential limitation is that we may have unintentionally included studies with populations offered antiviral medications during the study period if authors elected not to report antiviral use in their published manuscripts on influenza in LTCFs. The risk of including antiviral-exposed residents is especially high for studies of outbreaks, which are an indication for antiviral use. Including antiviral-exposed populations in our review would lead to an underestimation of the burden of infection. This concern is mitigated by research demonstrating that chemoprophylaxis with antivirals may be less effective for influenza prevention in unvaccinated residents of LTCFs [50].

A third potential limitation of our review was its focus on identifying the burden of respiratory infections among LTCF residents in the absence of intervention, particularly vaccination. This focus was necessary to understand the potential impact of new interventions and policies. Furthermore, many LTCF residents are unvaccinated and immunization rates remain unsatisfactorily low in LTCFs. This creates an essential need to understand the natural burden of respiratory infections and guide further prevention efforts as well as help make a more compelling argument for encouragement of vaccination by providers [51, 52]. However, since the use of vaccines has increased over time in LTCFs, our review may have unintentionally included an older evidence base that is less generalizable to contemporary LTCF residents. Related to this limitation is the challenge that many studies included populations of unvaccinated residents whose data was not presented separately from that of vaccinated residents. To remain within the scope of our review, we excluded studies that did not distinguish vaccinated and unvaccinated individuals. In doing so, we may have isolated a less generalizable evidence base.

An additional important potential limitation of our systematic review is that respiratory infections other than influenza, RSV, and pneumonia occur in LTCFs and can be important. We selected influenza, RSV, and pneumonia as the focus because of their clinical importance, but future reviews and research should consider other respiratory infections as well as the etiology and pathogens involved (i.e., specific organisms or strains). Other respiratory viruses of potential future interest should include adenovirus, coronavirus, metapneumovirus, parainfluenza virus, and rhinovirus. In older adults, respiratory viruses can also produce gastrointestinal symptoms, and therefore may go unrecognized as having a respiratory etiology. Given the breadth of respiratory infections and pathogens that are important in LTCFs, establishing a comprehensive surveillance program for the LTCF population may prove highly valuable for guiding clinical care and future interventions [53, 54].

## Conclusions

Limited data exist about the burden of respiratory infections among older adults in LTCFs. Most prior studies were not nationally representative, recent, or large enough to generate precise estimates. Well-designed studies are therefore needed to credibly identify the burden of respiratory infections in LTCFs. Furthermore, large and well-designed studies are still necessary to understand the determinants of increased respiratory infection risk in LTCFs as well as subsequent outcomes. Such data could ultimately help to reduce the burden of respiratory infections in LTCFs by informing intervention development, evidence-based clinical practice, and effective policymaking.

## Additional file

Additional file 1: **Table S1.** PubMed Search Strategy. **Table S2**. EMBASE search strategy. (DOCX 15 kb)

## Data Availability

Data sharing is not applicable to this article as no datasets were generated or analysed during the current study.

## Abbreviations

LTCF: long-term care facility
RCT: randomized controlled trial
RSV: respiratory syncytial virus
US: United States
