# Supplementary material for: The burden of respiratory infections among older adults in long-term care: a systematic review
Source: BMC Geriatr. 2019 Aug 5;19:210. doi: 10.1186/s12877-019-1236-6 (PMC6683564; doi:10.1186/s12877-019-1236-6)
Supplement: Supplementary file 1 — Table S1. PubMed Search Strategy. Table S2. EMBASE search strategy. (DOCX 15 kb) [file 12877_2019_1236_MOESM1_ESM.docx]

**Additional file 1: Table S1.** PubMed Search Strategy.

| **Respiratory Infection** | **Search Terms** | | | |
| --- | --- | --- | --- | --- |
| Influenza | "influenza, human"[MeSH Terms] OR ("influenza"[All Fields]) | "human"[All Fields]) OR "human influenza"[All Fields] OR "influenza"[All Fields]) | care[All Fields] OR home[All Fields] OR ("nursing"[Subheading] OR "nursing"[All Fields] OR "nursing"[MeSH Terms] OR "nursing"[All Fields] OR ("aged"[MeSH Terms] OR "aged"[All Fields] OR "elderly"[All Fields]) OR residential[All Fields] OR institutionalized[All Fields] OR institutionalised[All Fields] OR geriatric[All Fields] | NOT (("infant"[MeSH Terms] OR "infant"[All Fields] OR "infants"[All Fields]) OR ("child"[MeSH Terms] OR "child"[All Fields] OR "children"[All Fields]) |
| Pneumonia | "pneumonia"[MeSH Terms] OR "pneumonia"[All Fields] | "human"[All Fields]) OR "human influenza"[All Fields] OR "influenza"[All Fields]) | care[All Fields] OR home[All Fields] OR ("nursing"[Subheading] OR "nursing"[All Fields] OR "nursing"[MeSH Terms] OR "nursing"[All Fields] OR ("aged"[MeSH Terms] OR "aged"[All Fields] OR "elderly"[All Fields]) OR residential[All Fields] OR institutionalized[All Fields] OR institutionalised[All Fields] OR geriatric[All Fields] | NOT (("infant"[MeSH Terms] OR "infant"[All Fields] OR "infants"[All Fields]) OR ("child"[MeSH Terms] OR "child"[All Fields] OR "children"[All Fields]) |
| Respiratory Syncytial Virus | respiratory[All Fields] | "human"[All Fields]) OR "human influenza"[All Fields] OR "influenza"[All Fields]) | care[All Fields] OR home[All Fields] OR ("nursing"[Subheading] OR "nursing"[All Fields] OR "nursing"[MeSH Terms] OR "nursing"[All Fields] OR ("aged"[MeSH Terms] OR "aged"[All Fields] OR "elderly"[All Fields]) OR residential[All Fields] OR institutionalized[All Fields] OR institutionalised[All Fields] OR geriatric[All Fields] | NOT (("infant"[MeSH Terms] OR "infant"[All Fields] OR "infants"[All Fields]) OR ("child"[MeSH Terms] OR "child"[All Fields] OR "children"[All Fields]) |

**Table S2**. EMBASE search strategy

| **Respiratory Infection** | **Search Terms** | | | |
| --- | --- | --- | --- | --- |
| Influenza | Influenza | care OR home OR nursing OR elderly OR residential OR institutionalized OR institutionalized OR geriatric | NOT (infants OR children) | [humans]/lim |
| Pneumonia | Pneumonia | care OR home OR nursing OR elderly OR residential OR institutionalized OR institutionalized OR geriatric | NOT (infants OR children) | [humans]/lim |
| Respiratory Syncytial virus | ‘respiratory syncytial virus’ | care OR home OR nursing OR elderly OR residential OR institutionalized OR institutionalized OR geriatric | NOT (infants OR children) | [humans]/lim |
